# Occupational accidents involving biological material: demographic and occupational profile of affected workers

**DOI:** 10.47626/1679-4435-2020-534

**Published:** 2021-03-03

**Authors:** Caroline Bertelli, Bruna Rezende Martins, Suzane Beatriz Frantz Krug, Analídia Rodolpho Petry, Patrícia de Souza Fagundes

**Affiliations:** 1 Programa de Mestrado e Doutorado em Promoção da Saúde, Universidade de Santa Cruz do Sul (Unisc), Santa Cruz do Sul, RS, Brazil; 2 Centro Regional de Referência em Saúde do Trabalhador da Região dos Vales, Secretaria Municipal de Saúde de Santa Cruz do Sul, Santa Cruz do Sul, RS, Brazil

**Keywords:** exposure to biological agents, occupational health, occupational exposure, occupational accidents

## Abstract

**Introduction:**

Work accidents involving biological material are characterized as those whose exposure involves direct or indirect contact with human or animal blood and biological fluids, with a potential degree of contamination.

**Objectives:**

To investigate the sociodemographic and occupational profile of workers, as well as data on occupational accidents with exposure to biological material in the municipalities that make up the Centro de Referência em Saúde do Trabalhador da Região dos Vales do estado do Rio Grande do Sul (Cerest-Vales/RS).

**Methods:**

Documentary, retrospective, descriptive, quantitative research, where 1,260 Sistema de Informação de Agravos de Notificação (Sinan) notifications were analyzed, between 2014 and 2018. Data were collected in a unit specialized in worker health care.

**Results:**

The sex most affected by accidents was female, with 80.2% of cases, age group of 28 to 33 years (26.4%), and level of education represented mostly by complete high school (55.4%). Health professionals concentrated 84.1% of accidents, most of which occurred with nursing technicians, followed by nurses and doctors. Professionals from different occupations, such as veterinarians, students, janitors and garbage collectors were also exposed. Blood was the organic material that prevailed (81.1%) between accidents and exposure, percutaneous (70.3%). Clinical data revealed the prevalence of patients vaccinated for hepatitis B (90.6%), but 62.7% with (hepatitis B surface antigen, HBsAg) negative; 49.5% of the injured were discharged with a negative source patient and 66% the Comunicação de Acidente de Trabalho (CAT) issued.

**Conclusions:**

Accidents occurred more frequently among females, individuals with low education and health professionals. The weakness in the reports of accidents with professionals with no presumed risk is reiterated, which generates underreporting rates.

## INTRODUCTION

Work is a major component of individual identity and plays an important role in improving living conditions, supporting families and shaping the global economy. Yet work can also cause exposure to occupational risk factors, which have a negative impact on health and contribute to the development of several diseases.^1,2^ Occupational accidents (OAs) are single events, limited in space and time, which occur as a consequence of occupational activities and result in physical harm or functional impairment. Their repercussions are usually immediate and can include death or work-related disability (temporary or permanent, total or partial).^3^ Incidents that involve direct or indirect contact with contaminants such as human or animal blood or bodily fluids constitute a specific type of OA, referred to as an accident involving exposure to biological material (AEBM). These incidents are usually caused by needles, sharp instruments or the exposure of skin or mucous membranes.^4,5^

AEBM leave workers vulnerable to contamination by more than 60 pathogen species, three of which have great epidemiological relevance: the human immunodeficiency virus (HIV), the hepatitis B (HBV) and the hepatitis C virus (HCV). Health care workers are especially susceptible to AEBM. However, these incidents can also affect workers in other occupational sectors, including funeral services, the beauty industry, public safety, and sanitation.^2,4,6^

In Brazil, AEBM must be reported to the competent authorities. According to ordinance No. 104, issued January 25, 2011, these and ten other types of incident must be reported to the Information System for Notifiable Diseases (Sinan). The high incidence of AEBM in Brazil has been identified as a major area of concern by the national Ministry of Health.^7,8^ Brazil recently ranked fourth out of 200 countries in terms of the frequency of fatal occupational injuries, with the top three spots occupied by the United States, Thailand and China.^9^ From 2010 to 2015, the Sinan received 809,520 reports of occupational illness and injury, with 276,699 (34,2%) of these cases involving biological material.^10^ Also, data provided by the General Coordination of Occupational Health - CGST directly to the authors by email, revealed that between the years 2015 to 2018, 232,899 cases of ATMB were notified, part of them located in the state of Rio Grande do Sul, state which totaled 15,616 situations.

AEBM represent a serious public health problem, and affect primarily young, working-age adults; as such, specialized monitoring systems such as the Sinan emerge as important tools for epidemiological surveillance and data collection.^11,12^ Specialized services such as Occupational Health Centers (Cerest) also make a direct contribution to the reporting of AEBM.^13^

Though this issue has been examined by a vast number of studies, most of the literature on AEBM focuses specifically on health care workers.^4,14^ Few investigations have examined this type of incident across different occupations, highlighting the need for the present study. By collecting and analyzing data on AEBM, we intend to provide a broader perspective on this issue, identifying potential interventions to reduce the occurrence of these incidents. The aim of this study was therefore to investigate the sociodemographic and occupational characteristics of workers involved in AEBM, and characterize the incidents that have taken place in the cities served by the Occupational Health Center in the Valley Region of the state of Rio Grande do Sul (Cerest-Vales/RS).

## METHODS

This was a documentary, retrospective, descriptive, quantitative study based on the analysis of secondary data from a specialized occupational health service - Cerest-Vales/RS - in the city of Santa Cruz do Sul (RS). The six health regions served by the Cerest-Vales include 68 cities with a total of 899,833 inhabitants. According to the 2016 Annual Social Information Report (RAIS), the region is also home to 26,050 formal work organizations, including companies, city halls and other businesses. From 2014 to 2018, a total of 33,066 incidents in the 68 cities were reported to the Sinan and the Occupational Health Information System (SIST). The present study retrieved and analyzed all incident reports filed between January 1, 2014, and December 31, 2018, in the Sinan-Net database.

The electronic records of 1,266 AEBM were obtained from the Cerest-Vales itself, which retrieved the data from the Health Surveillance Center (CEVS) of the state of Rio Grande do Sul. Inclusion criteria consisted of incidents involving workers aged 16 years or older, whether students or employees in the formal and informal sectors, within the area served by the Cerest-Vales. Records where the ‘occupation’ field was left blank were excluded from the study. This led to the exclusion of six records from the initial sample, leaving a total of 1,260 incidents for analysis.

This study was conducted in accordance with the guidelines in Resolution 466/2012, and was approved by the Research Ethics Committee of the Universidade de Santa Cruz do Sul (Unisc), under protocol number 3.466.277 and CAAE 16976819.3.0000.5343. The variables analyzed in this study included sociodemographic characteristics (gender, age and education), occupational factors (occupation, type of work contract, and length of current employment), features of the incident itself and clinical outcome following exposure (type of exposure, type of biological material, object, use of personal protective equipment [PPE], vaccination status, case evolution and publication of Occupational Accident Report [CAT]).

Workers were categorized by type of occupation and similarity of qualifications, and job titles were coded according to the Brazilian Classification of Occupations (CBO). As such, high-level health care professionals were categorized by occupation, and technical workers were divided into nursing technicians and other health care technicians. A similar process was used to classify the remaining professions according to educational background. All data were entered into Microsoft Excel and exported to SPSS version 20.0 for descriptive analysis of frequencies and percentages.

## RESULTS

From 2014 to 2018, 1,266 AEBM were reported to the authorities. Six (0.5%) of these incidents were excluded from analysis since the ‘occupation’ field was left blank, which was an exclusion criterion for this study. This resulted in a total sample of 1,260 reports. The sociodemographic data in Table 1 shows that most workers who experienced AEBM were in the 28- to 33-year-old age group, which comprised 333 (26.4%) participants. A total of 1,010 (80.2%) incidents involved women while 250 (19.8%) involved men, and most workers had completed secondary education, as observed in 698 (55.4%) cases.

**Table 1:** Sociodemographic characteristics of workers who had accidents involving exposure to biological material, 2014-2018

Variable	n (%)
Gender	
Female	1,010 (80.2)
Male	250 (19.8)
Total	2,260 (100.0)
Age (years)	
16-21	118 (9.4)
22-27	314 (24.9)
28-33	333 (26.4)
34-39	226 (180.0)
40-45	126 (10.0)
46-51	67 (5.3)
52-57	38 (3.0)
58-63	25 (2.0)
Over 63	4 (0.3)
No data	9 (0.7)
Total	1,260 (100.0)
Education	
Incomplete primary	34 (2.7)
Complete primary	16 (1.3)
Incomplete secondary	33 (2.6)
Complete secondary	698 (55.4)
Incomplete higher education	97 (7.7)
Complete higher education	248 (19.7)
Missing/blank	134 (10.6)
Total	1,260 (100.0)

Source: Information System for Notifiable Diseases (2019).

Table 2 describes the occupations of workers with AEBM within the study period. While 1,060 (84.1%) of these individuals worked in health care or related fields, 200 (15.9%) incidents involved workers in other areas. Most reports involved mid-level health professionals, with nursing technicians accounting for 60.1% of the cases analyzed. Higher-level health care professionals, in turn, accounted for 238 (18.8%) cases.

**Table 2:** Occupations of accident victims, 2014-2018

Variable	n (%)
Mid-level health professionals	
Nursing technicians	757 (60.1)
Higher-level health professionals	
Nurses	116 (9.3)
Physicians	80 (6.4)
Dentists	28 (2.3)
Pharmacists	12 (10)
Other	4 (0.3)
Other technical health professions	
Pharmacy technicians	23 (1.8)
Dental assistants and hygienists	13 (1.0)
Nursing and radiology assistants and technicians	5 (0.4)
Surgical technicians	19 (1.5)
Other higher-level professions	
Veterinarians	5 (0.4)
Administrators	3 (0.2)
Other	
Students	56 (4.5)
Janitors	24 (1.9)
Garbage collectors and elementary occupations	22 (1.7)
Other^*^	19 (1.5)
Domestic workers and building cleaners	14 (1.1)
Laundry workers	10 (0.8)
Members of the Armed Forces, police officers and firefighters	9 (0.7)
Beauty service providers	8 (0.6)
Automobile, truck and motorcycle drivers	9 (0.7)
General maintenance workers	5 (0.4)
Occupational and environmental health inspectors	6 (0.5)
Administrative assistants	4 (0.3)
Customer service workers	4 (0.3)
Agricultural and livestock technicians	3 (0.2)
Chronically unemployed	2 (0.1)
Total	1,260 (100.0)
Type of work contract	
Full-time contract	960 (76.2)
Statutory and contracted public servants	113 (9.0)
Other	68 (5.4)
Independent/Self-employed	66 (5.2)
Unregistered worker	23 (1.8)
Missing/blank	14 (1.1)
Cooperative worker	10 (0.8)
Temporary workers and independent contractors	6 (0.5)
Total	1,260 (100.0)
Length of current employment (years)	
Less than 1	313 (24.8)
1-2	252 (20.0)
3-4	152 (12.1)
5-6	115 (9.1)
7-10	87 (6.9)
Over 10 years	128 (10.2)
Missing	213 (16.9)
Total	1,260 (100.0)

Source: Information System for Notifiable Diseases (2019).

* Single incidents involving funeral service workers, doormen, security guards, gas station attendants, butchers and production line workers.

In addition to higher-level workers in the health care sector, the sample included five veterinarians and three administrators. As can be seen in Table 2, students accounted for 4.4% of cases, while janitors were involved in 1.9% of incidents. Garbage collectors and other elementary occupations accounted for a similar percentage of cases (1.7%). A small number of customer service workers and agricultural or livestock technicians was also present in the sample, accounting for three and two incidents, respectively. Table 2 shows that most accident victims were contract workers, as observed in 76.2% of cases, while 9% of incidents involved statutory and contracted public servants.

A significant proportion of workers (24.8%) had been in their current job for less than one year at the time of the accident. However, this field was left blank in a large number of records, so that data were missing in 213 (16.9%) cases. The data in Table 3 shows that most cases (70.3%) involved percutaneous exposure incidents. The majority of events also involved blood exposure, as reported in 81.8% of incidents. A relevant number of cases (102, 8.1%) were also classified as “Other” (single cases involving vomit, semen and other fluids such as sputum, urine and gastric contents).

**Table 3:** Data regarding occupational exposure incidents, 2014-2018

Variable	n (%)
Type of exposure	
Percutaneous	886 (56.7)
Intact skin	383 (24.5)
Mucous membrane	193 (12.3)
Non-intact skin	85 (5.5)
Other	15 (10)
Total	1,562 (100.0)
Type of fluid	
Blood	1,022 (81.1)
Other	102 (8.1)
Bloody fluid	59 (4.7)
Missing	41 (3.2)
Cerebrospinal fluid	15 (1.2)
Blank	11 (0.9)
Ascitic or amniotic fluid	5 (0.4)
Serum/plasma	5 (0.4)
Total	1,260 (100.0)
Object	
Hollow-bore needle	647 (51.4)
Other	338 (26.8)
Solid needle	154 (12.2)
Blade/lancet	83 (6.6)
Missing/blank	23 (1.8)
Intracath needle	9 (0.7)
Glassware	6 (0.5)
Total	1,260 (100.0)
PPE use	
Gloves	852 (44.1)
Gown	491 (25.4)
Protective eyewear	249 (12.9)
Mask	199 (10.3)
Boots	121 (6.3)
Facial protection	20 (10)
Total	1,932 (100.0)

PPE = personal protective equipment.

Source: Information System for Notifiable Diseases (2019).

The objects most frequently involved in the incidents were hollow-bore needles, which accounted for 647 incidents, followed by those in the “Other” category (338, 26.8%) and solid needles (n = 154, 12.2%). The type of PPE most commonly used by participants at the time of the accident were gloves (n = 852, 67.6%), while the least commonly used were facial protection equipment (20, 1.6%). The data from 2014 to 2018 also revealed that most of the incidents in the region served by the Cerest occurred in five cities, which accounted for 68.8% of events. Sixteen (23.5%) of the cities in the catchment area did not report any incidents during the study period.

Table 4 describes the clinical outcomes of workers after the accidents. The data show that 3% of individuals had not been vaccinated for hepatitis B, increasing the likelihood of contracting the illness. Further analysis showed that seven individuals (0.5%) tested positive for the hepatitis B surface antigen (HBsAg) while 325 (25.8%) were negative for anti-HBs. The latter finding suggests that many of the workers who had AEBM did not have antibodies against the disease, despite receiving all three doses of the HBV vaccine. Most participants were also negative for anti-HCV antibodies, as observed in 820 (65.1%) cases. Our findings also showed that eight (0.6%) individuals tested positive for HIV on rapid tests performed after their respective incidents. Table 5 shows the evolution of cases over time. The results show that 624 (49.5%) of the 1,260 workers who had AEBM were discharged after the source of exposure tested negative for HIV, while 341 (27.1%) were discharged with no serological conversion. A CAT was issued in 832 (66%) cases after the accidents. It is also important to note that 76.2% of workers in the sample were formally employed.

**Table 4:** Clinical data on workers exposed to biological materials, 2014-2018

Variable	n (%)
Hepatitis B vaccination status	
Positive	1,142 (90.6)
Missing	66 (5.2)
Negative	38 (3.0)
Blank	14 (1.2)
Total	1,260 (100.0)
Anti-HCV	
Positive	0 (0.0)
Negative	820 (65.0)
Inconclusive	6 (0.5)
Not tested	307 (24.4)
Missing	75 (6.0)
Blank	52 (4.1)
Total	1,260 (100.0)
Anti-HIV	
Negative	897 (71.2)
Not tested	242 (19.2)
Missing	61 (4.8)
Blank	45 (3.6)
Positive	8 (0.6)
Inconclusive	7 (0.6)
Total	1,260 (100.0)
HBsAg	
Negative	790 (62.7)
Not tested	334 (26.5)
Missing	73 (5.8)
Blank	50 (4.0)
Positive	7 (0.5)
Inconclusive	6 (0.5)
Total	1,260 (100.0)

HBsAg = hepatitis B surface antigen; HCV = hepatitis C virus; HIV = human immunodeficiency virus.

Source: Information System for Notifiable Diseases (Sinan) (2019).

**Table 5:** Case evolution, 2014-2018

Variable	n (%)
Case evolution	
Patient discharged - source negative	624 (49.5)
Patient discharged - no serological conversion	341 (27.1)
Blank/missing	244 (19.4)
Patient discharged - with serological conversion	34 (2.7)
Drop-out	17 (1.3)
Total	1,260 (100.0)
CAT issued	
Yes	832 (66.0)
No	195 (15.5)
Not applicable	25 (2.0)
Missing/blank	208 (16.5)
Total	1,260 (100.0)

CAT =  occupational accident report.

Source: Information System for Notifiable Diseases (2019).

## DISCUSSION

The first important observation made in the present study was that the ‘occupation’ field was left blank in six (0.5%) of the incidents analyzed. Similar findings were obtained in a descriptive study conducted in the city of Betim, in the state of Minas Gerais, which evaluated the completeness of Sinan records for all 11 types of incident reports, and found that the ‘occupation’ field was left blank in 5% of cases.^15^ One explanation for the missing data in these reports pertains to the culture of each occupation and the difficulties experienced in the reporting process.^16^ The high rates of underreporting and missing data interfere with the accuracy of the records, preventing institutions from developing and implementing procedures to foster a culture of preventive practice, characterized by specific and effective strategies to reduce accident numbers. It is also important to note that many other items in the Sinan records were either missing or blank. The Centers for Disease Control and Prevention (CDC) in the United States evaluates data quality based on the percentage of “unknown” or “blank” responses, presence of duplicate information and consistency of health information systems, all of which are then used to determine the accuracy with which the records represent the phenomenon of interest.^15^ Such methods are crucial for personnel qualification and the constant improvement of surveillance systems.

Most of the incidents analyzed in the present study involved young workers, aged 28 to 33 years. These findings are similar to those of a previous study which analyzed the occurrence of AEBM in Brazil over a 7-year-period, and found that the most affected age group was that of 25- to 29-year-olds.^4^ A higher prevalence of occupational injuries in younger workers relative to their older peers has been consistently reported in the literature, suggesting that these incidents may be related to inexperience and a lack of technical knowledge, possibly due to limitations of their educational background.^17,18^ Most of the incidents involved female workers, which also corroborates previous studies, and is explained by the fact that the occupations analyzed, especially in the health care sector, are predominantly held by women.^19^

The most frequent education level in the sample was secondary school. These findings are similar to those of a study conducted in the state of Minas Gerais, where 32.4% of participants had the same education level. These results suggest that health care professions in Brazil are predominantly performed by individuals with only secondary education. This may be attributed to the lower cost of hiring technical workers, but also reflects the low education levels of the Brazilian population as a whole.^4,15^ The present study revealed several occupations in which AEBM are especially common; though most incidents affected health care workers, many other professionals are also vulnerable to this type of accident.

The fact that health care workers provide uninterrupted assistance to patients, come into direct contact with disease, handle biological material and carry out several health care procedures makes them especially susceptible to occupational accidents.^15,19^ In Brazil, a total of 331,603 cases of AEBM were reported to the Sinan from 2010 to 2016. Health care workers accounted for 243,621 (73.4%) of these cases. These figures are similar to those observed in the present study, where 84.1% of cases involved health care professionals.^16^ It is also likely that incident reporting behaviors are far more central to the occupational culture in health care professions than other occupations, contributing to discrepancies in accident rates.

A significant number of students were also affected by AEBM. Studies suggest that this type of incident is common in academic settings due to the age and inexperience of students, who are unfamiliar with the execution and development of procedures involving biological materials.^20^ It is estimated that 15 million people worldwide work in the collection of recyclable materials. In Brazil, approximately 1 million workers are active in this industry. These workers are also affected by the inadequate disposal of biological waste.^14,21,22^ Cleaning and sanitation workers are also exposed to AEBM, as was verified in the present study. The severity of the incidents involving these workers is aggravated by the fact that the materials to which they are exposed often come from unknown sources, leading to more serious injuries.^20^

The present study also found that the occupational activities of beauty industry workers carry the risk of exposure to blood-borne pathogens. The lack of knowledge and adherence to biosecurity measures, such as hand hygiene, instrument reprocessing practices and disposal of single-use items are known issues in this industry.^22^ In the state of Minas Gerais, a study of 54 manicurists and pedicurists found that 31.5% of these professionals had been injured by sharp instruments between 2010 and 2011, with most injuries (76.5%) caused by eponychium (cuticle) trimmers. These professionals can be exposed to microorganisms through direct or indirect percutaneous or mucosal contact, as well as contact with intact or non-intact skin.^4,23^ Minor cuts on the skin surface caused by nail clippers, tattoo needles, shaving razors, scissors and other sharp instruments can cause trauma or microtrauma that facilitates the transmission of blood-borne pathogens between clients and professionals. Such incidents, combined with the sharing of sharp instruments with relatives and friends at home and in beauty parlors, have been found to increase the risk of horizontal pathogen transmission.^24^

Beauty parlors are directly responsible for disposing of the waste generated by their activities due to the high risk of OAs and infectious diseases. These establishments are therefore responsible for ensuring proper waste disposal and contributing to a culture of prevention.^21,24^ The occurrence of AEBM involving veterinarians and animal health professionals was analyzed in a study of victims of AEBM in Brazil between 2007 and 2014; like the present investigation, the aforementioned study found that veterinarians were among the higher-level occupations most affected by this type of accident, with an incident rate of 17.9 per 1.000 workers/year.^4^ Additional occupations with a high risk of AEBM are members of the armed forces, police officers and firefighters, especially during military activities, combat or humanitarian operations.^4^

In the present study, blood was the most common contaminant in the incidents reported, and percutaneous exposure was the most frequent accident type. This finding is in line with that of a descriptive study of health services in Boa Vista, city of the state of Roraima, where 78.8% of accidents involved percutaneous exposure and 76% of cases involved accidental exposure to blood.^14^ Percutaneous needle exposure accounts for 80 to 90% of infectious diseases in health care professionals. The risk of HBV transmission after such an accident is 1 in 3, while the risk of HCV transmission is one in 30 and that of HIV, 1 in 300. It is important to note that HBV is highly resistant to the environment, surviving for up to one week in dried blood on external surfaces.^24,25^ The analysis of PPE use revealed that in 32.4% of accidents, the affected individuals were not wearing gloves. This finding is in line with that of a study conducted in Minas Gerais, which found that 35.7% of accident victims were not wearing gloves at the time of exposure. Additionally, much like the present findings, the aforementioned study found that facial protection was not worn in 88.3% of cases.^26^

The use of PPE is related to workers’ perceived risk of occupational exposure to contaminants. Yet according to standard precautions, this risk is universal, and any individual can potentially transmit infectious microorganisms. PPE use and hand hygiene practices should therefore be adopted by all professionals, regardless of perceived contamination risk.^16,18^ The present study revealed that 76.2% of individuals were formally employed, though a CAT was only issued in 66% of cases. This finding underscores the problem of underreporting in the formal employment sector, since this document is only issued for workers covered by Work Accident Insurance (SAT)^27^. Workers exposed to this type of accident may suffer physical injuries and have high treatment costs, in addition to psychological issues such as emotional distress due to the possibility of contamination and transmission of illnesses to relatives.^28^

## CONCLUSIONS

This study demonstrated that females and workers with a low education level are especially affected by AEBM. Most reports involved health care professionals, followed by occupations such as veterinarians and garbage collectors. It is important to note that many professionals were not immune to HBV, raising the risk of infection.

The higher rate of incidents among health care professionals also reflects a cultural or behavioral propensity to report these events. Professionals with low risk perception may fail to identify AEBM, and are therefore unlikely to seek medical attention which would prompt a notification, increasing the rates of underreporting and contributing to a growing public health problem. It must be noted that the reporting of incidents to the Sinan was originally intended to be the responsibility of health care providers. This is a limiting factor that must be discussed, evaluated and addressed. Another limitation of this study is that it is one of the first to examine this issue; the fact that most of the literature on AEBM focuses on health care workers limits the discussion of the present findings.
